# European ST80 community-associated methicillin-resistant *Staphylococcus aureus* orbital cellulitis in a neonate

**DOI:** 10.1186/1471-2415-12-7

**Published:** 2012-04-10

**Authors:** Evangelia E Tsironi, Fani Zacharaki, Ioanna N Grivea, Sophia V Tachmitzi, Aspasia N Michoula, Marianna Vlychou, Efthimia Petinaki, George A Syrogiannopoulos

**Affiliations:** 1 Department of Ophthalmology, University General Hospital of Larissa, University of Thessaly, Medical School, Biopolis, Larissa, 41110, Greece; 2 Department of Pediatrics, University General Hospital of Larissa, University of Thessaly, Medical School, Larissa, Greece; 3 Department of Radiology, University General Hospital of Larissa, University of Thessaly, Medical School, Larissa, Greece; 4 Department of Microbiology, University General Hospital of Larissa, University of Thessaly, Medical School, Larissa, Greece

**Keywords:** Neonatal orbital cellulitis, Methicillin-resistant, *Staphylococcus aureus*, Daptomycin

## Abstract

**Background:**

Methicillin-resistant *Staphylococcus aureus* is a serious cause of morbidity and mortality in hospital environment, but also, lately, in the community. This case report is, to our knowledge, the first detailed description of a community-associated methicillin-resistant *S. aureus* ST80 orbital cellulitis in a previously healthy neonate. Possible predisposing factors of microbial acquisition and treatment selection are also discussed.

**Case presentation:**

A 28-day-old Caucasian boy was referred to our hospital with the diagnosis of right orbital cellulitis. His symptoms included right eye proptosis, periocular edema and redness. Empirical therapy of intravenous daptomycin, rifampin and ceftriaxone was initiated. The culture of pus yielded a methicillin-resistant *S. aureus* isolate and the molecular analysis revealed that it was a Panton-Valentine leukocidine-positive ST80 strain. The combination antimicrobial therapy was continued for 42 days and the infection was successfully controlled.

**Conclusions:**

Clinicians should be aware that young infants, even without any predisposing condition, are susceptible to orbital cellulitis caused by community-associated methicillin-resistant *S. aureus.* Prompt initiation of the appropriate empirical therapy, according to the local epidemiology, should successfully address the infection, preventing ocular and systemic complications.

## Background

Over the past 2 decades, the incidence of community-associated methicillin-resistant *Staphylococcus aureus* (CA-MRSA) infections has constantly been rising, due to the emergence of highly virulent and transmissible strains [1]. In a recent study performed in our area, CA-MRSA was found to be increasing as a cause of skin and soft tissue infections, as well as of the invasive ones, among all pediatric ages [2]. Fortunov et al. have reported that CA-MRSA infections are also increasing in previously healthy neonates without traditional risk factors and males are most often affected between 7 to 12 days of age [3,4].

Greece is a country with increased incidence of CA-MRSA. This expansion is associated mainly with the wide spread in the community of a single virulent clone, the European ST80. In our area, the proportion of staphylococcal pediatric infections caused by a CA-MRSA isolate increased from 51.5% in 2003-2006 to 63.4% in 2007-2009 [2]. In addition, an increasing rate of resistance to clindamycin has been noted. The rate of clindamycin resistance in MRSA isolates between 2007 and 2009 has been 23.8-31.2% and that of MSSA isolates 15.4-38.1% [2]. Currently in central Greece, vancomycin, linezolid, or daptomycin [5,6], as monotherapy or in combination with rifampin or gentamicin, appear to be choices for empirical therapy of severe possible staphylococcal pediatric infections.

Cases of CA-MRSA periocular infections in pediatric patients have been reported, the most recent being a report of two cases of infantile CA-MRSA orbital cellulitis by Kobayashi et al. [7]. Kodsi reported a case of chronic dacryocystitis due to CA-MRSA in an 8.5-month-old boy with congenital nasolacrimal duct obstruction [8]. According to the author, this child's infection may have been related to chronic oral antibiotics administered since birth to prevent urinary tract infections related to hypospadias.

Another case of perinatal CA-MRSA dacryocystitis and periorbital cellulitis in a 12-day-old previously healthy neonate has recently been reported [9]. In this patient, vertical transmission of MRSA may have occurred during vaginal delivery or subsequent close contact, including breast feeding.

Mother, during or after delivery, family members or birth in the hospital may be implicated in CA-MRSA acquisition. An interesting study of more than 5,000 pregnant women proved that 3.5% of them had vaginal colonization with MRSA. There was no evidence of MRSA disease among their newborn children [10]. These findings, however, suggest that vertical transmission of MRSA is theoretically possible during vaginal delivery.

A recent study of Fortunov et al. revealed that healthy term and late preterm neonates were more often infected by *S. aureu*s with their own nasal strain than with their mother's nasal strain [11].

In order to highlight the wide age and clinical manifestation spectrum of CA-MRSA infections, we report a case of perinatally acquired MRSA orbital cellulitis.

## Case presentation

Our patient, a 28-day-old boy, was referred to the Department of Pediatrics of our hospital with the diagnosis of right-sided orbital cellulitis on April 28, 2010. His symptoms, including anorexia, rhinorrhoea, and right eye redness had begun five days before admission.

The newborn was full-term and had been delivered vaginally. Birth weight was 4,900 g and he was on combined breast and formula feeding pattern.

On admission, the patient's temperature was 37.7°C, heart rate was 132 beats/min, and physical examination revealed no other pathological findings except right eye proptosis, conjunctival injection and periorbital erythema. The infant did not have evidence of pneumonia on chest radiograph.

White blood cell count was 19,000 cells/mm^3^ with differential of neutrophils 49%, lymphocytes 24%, monocytes 11%, activated lymphocytes 10%, myelocytes 1% and eosinophils 5%. Platelet count was 373,000/mm^3^. Blood, urine, and cerebrospinal fluid (CSF) samples were cultured and proved sterile. The examination of CSF showed 300 RBC/mm^3^, 2 WBC/mm^3^, glucose 3.88 mmol/L with concomitant blood glucose 6.55 mmol/L, protein 0.52 g/L and LDH 24 IU/L. Erythrocyte sedimentation rate (ESR) was 91 mm/h and C-reactive protein (CRP) was 120 mg/L.

Ophthalmic examination revealed right eye proptosis, periocular redness and edema and conjunctival discharge. Ocular motility was severely impaired. Pupils were equal, round, normally reactive to light. Slit-lamp anterior segment examination and dilated fundus examination were within normal limits.

**Figure 1:**
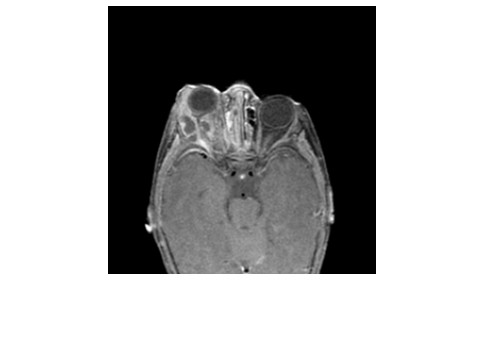
**Initial Orbit MRI.** Axial T1 post Gd MR image shows unilateral exophthalmos of the right eye due to inflammatory collections with central liquification and peripheral enhancement suggestive of a multiloculated abscess that occupy the inferior and posterolateral aspect of the orbit. The adjacent ethmoid sinuses are extensively mucus-filled with heterogeneous enhancement, consistent with sinusitis.

Brain MRI was normal and orbital MRI at 3.0T showed ethmoid opacification and a right eye proptosis due to an intra-orbital lesion, located in the retrobulbar fatty tissue between the medial, lateral, inferior rectus muscles and the wall of the globe. The lesion, containing multiple cystic regions, was consistent with orbital cellulitis with abscess formation (Figure1).

Empirical therapy of intravenous daptomycin 12 mg/kg qd, rifampin 20 mg/kg qd and ceftriaxone 100 mg/kg qd was initiated. On the fifth day of treatment, as the abscess had expanded to the preseptal area and the right eye proptosis continued with tension and edema of the lower eyelid, surgical drainage was performed through the inner area of the lower fornix. The culture of pus yielded a low colony count of MRSA. Polymerase chain reaction was performed in order to test the isolate for genes encoding the Panton-Valentine leukocidine (PVL) production (*luk*-*S*-*PV* and *luk*-*F*-*PV*) as previously described [2]. In addition, the *S. aureus* strain was studied by multilocus sequence typing. The molecular analysis of the isolate revealed that it was a PVL-positive ST80 *S. aureus* strain. The isolate was resistant to oxacillin, tetracycline and fucidic acid and susceptible to erythromycin, trimethoprim/sulfamethoxazole, vancomycin, linezolid, daptomycin, rifampin and moxifloxacin. The MIC to daptomycin was determined by the E-test method (AB Biodisk, Solna, Sweden) and was found to be 0.19 mg/L.

The infant remained afebrile. Further drainage of a small quantity of pus from the previously incised area was performed on the sixth day of treatment. Orbital MRI was performed the following day, revealing reduction of the abscess cavity volume, particularly at the posterolateral aspect of the orbit.

ESR was 40 mm/h and CRP was 3 mg/L on the eighth day of treatment and on the 15th day, ESR was 21 mm/h and CRP decreased to 0.1 mg/L.

During the following weeks, his ophthalmic findings were gradually improved without requirement for further surgery. The edema of the lower eyelid subsided fully during the fourth week of treatment. A repeat MRI was performed after 4 weeks of antimicrobial therapy, in which residual inflammatory findings were observed in the inferolateral structures of the orbit.

The combination antimicrobial therapy was administered for 42 days and successfully controlled the infection. The patient tolerated well the 6-week daptomycin therapy. The infant had normal creatinine and blood urea nitrogen values. No increase in serum creatine phosphokinase (CPK) levels was noted. CPK level was measured at least once per week and it ranged from 31 to 113 IU/L (median value 87 IU/L). A follow-up evaluation was performed 4 weeks after the completion of antimicrobial therapy and both the clinical examination and MRI findings were normal without residual ophthalmic disease. Since then, no relapse or long-term ophthalmic sequelae have been observed.

Maternal medical history and the thorough clinical investigation failed to reveal obvious predisposing factors of CA-MRSA acquisition or the site of entrance.

## Conclusions

Clinicians should be aware that young infants, even without any predisposing condition, are susceptible to orbital cellulitis caused by CA-MRSA. Moreover, in these cases the way of microbial acquisition or the site of entrance may remain unclear.

In a serious infection, such as orbital cellulitis, the following indications should be carefully considered as to when to initiate empirical treatment against CA-MRSA: (1) clinical suspicion or imaging findings consistent with orbital abscess; (2) extreme ages, such as infants under 3 months of age, especially during the neonatal period; and (3) an area where MRSA infections are not rare (prevalence of >5%).

In areas like our geographic region, where European ST80 CA-MRSA is endemic as a cause of skin and soft tissue as well as of invasive infections, empirical therapy with agents active against CA-MRSA should be initiated immediately in cases of orbital cellulitis.

### Consent

Written consent was obtained from the patient's parents for publication of this material. A copy of the consent is available for review.

## Competing interests

The authors declare that they have no competing interests relevant to this article to disclose.

## Authors' contributions

EET was the main physician responsible for the patient, performed the abscess drainage and revised the manuscript, FZ performed manuscript writing, ING, SVT and ANM managed and followed the patient, EP performed the molecular analysis of the bacterial strain, MV performed MRI imaging, and GAS managed the patient and helped to draft the manuscript. All authors read and approved the final manuscript.

## Pre-publication history

The pre-publication history for this paper can be accessed here:

http://www.biomedcentral.com/1471-2415/12/7/prepub
